# A Comparative Analysis of the Chemical Composition, Anti-Inflammatory, and Antinociceptive Effects of the Essential Oils from Three Species of *Mentha* Cultivated in Romania

**DOI:** 10.3390/molecules22020263

**Published:** 2017-02-10

**Authors:** Cristina Mogosan, Oliviu Vostinaru, Radu Oprean, Codruta Heghes, Lorena Filip, Georgeta Balica, Radu Ioan Moldovan

**Affiliations:** 1 Department of Pharmacology, Physiology and Physiopathology, Iuliu Hatieganu University of Medicine and Pharmacy, Cluj-Napoca 400349, Romania; cmogosan@umfcluj.ro; 2 Department of Analytical Chemistry, Iuliu Hatieganu University of Medicine and Pharmacy, Cluj-Napoca 400349, Romania; roprean@umfcluj.ro; 3 Department of Drug Analysis, Iuliu Hatieganu University of Medicine and Pharmacy, Cluj-Napoca 400349, Romania; cmaier@umfcluj.ro; 4 Department of Bromatology, Iuliu Hatieganu University of Medicine and Pharmacy, Cluj-Napoca 400349, Romania; lorenafilip@yahoo.com; 5 Department of Pharmaceutical Botany, Iuliu Hatieganu University of Medicine and Pharmacy, Cluj-Napoca 400337, Romania; bgeorgeta@umfcluj.ro; 6 SC Fares Biovital Laboratories SRL, Orastie 335700, Romania; cercetare@fares.ro

**Keywords:** *Mentha* spp., menthol, carvone, piperitenone oxide, anti-inflammatory, antinociceptive

## Abstract

This work was aimed at correlating the chemotype of three *Mentha* species cultivated in Romania with an in vivo study of the anti-inflammatory and antinociceptive effects of essential oils. The selected species were *Mentha piperita* L. var. *pallescens* (white peppermint), *Mentha spicata* L. subsp. *crispata* (spearmint), and *Mentha suaveolens* Ehrh. (pineapple mint). Qualitative and quantitative analysis of the essential oils isolated from the selected *Mentha* species was performed by gas chromatography coupled with mass spectrometry (GC-MS). The anti-inflammatory activity of the essential oils was determined by the rat paw edema test induced by λ-carrageenan. The antinociceptive effect of the essential oils was evaluated by the writhing test in mice, using 1% (*v*/*v*) acetic acid solution administered intraperitonealy and by the hot plate test in mice. The results showed a menthol chemotype for *M. piperita pallescens*, a carvone chemotype for *M. spicata,* and a piperitenone oxide chemotype for *M. suaveolens*. The essential oil from *M. spicata* L. (EOMSP) produced statistically significant and dose-dependent anti-inflammatory and antinociceptive effects.

## 1. Introduction

The genus *Mentha* (Lamiaceae), present in the temperate regions of all five continents, consists of 18 species and 11 named hybrids [1]. The members of the genus *Mentha* can easily produce many intermediary forms by hybridization, polyploidy playing also an important role in the speciation, making the number of taxonomically valid species a subject of controversy [2]. *Mentha* species are characterized by high morphological variability but also by a great chemical diversity with respect to their essential oils, the main chemical constituents, rarely encountered in other temperate zone species [3]. The differences in essential oil composition among the members of this genus offer a variety of strains with high contents of menthol, menthone, carvone, linalool, or other valuable terpenoid components synthesized by the mevalonic acid pathway [4]. Essential oils from *Mentha* species are widely used in food and beverage or cosmetic industries due to their flavoring properties [5]. Since ancient times, *Mentha* species have been used in traditional medicine for their carminative and antispasmodic properties in a variety of disorders of the gastro-intestinal tract or cholecystopathies [6]. More recently, several pre-clinical studies which investigated the pharmacological effects of the active constituents from *Mentha* species, demonstrated significant antimicrobial, antifungal, and antiviral activities [7,8,9], strong antioxidant and anticancer actions [10,11], but also antinociceptive, anti-inflammatory, and antiallergic properties [12,13,14]. Thus, evidence-based research demonstrated that *Mentha* species can be used as complementary or alternative remedies in a variety of pathologic conditions [15]. Due to their economic importance, extended cultures of *Mentha* species can be found in various climatic regions, in Europe, North-America or Asia [16].

In Romania, three species of *Mentha* are frequently cultivated. *Mentha piperita* L. var. *pallescens* (white peppermint) was among the first cultivated medicinal and aromatic plant, an experimental culture being created in 1908 [17]. *Mentha spicata* L. subsp. *crispata* (spearmint) is a creeping rhizomatous and perennial herb present in the spontaneous flora from the Balkan Peninsula but also under cultivation. *Mentha suaveolens* Ehrh. var. *variegata* (pineapple mint) is used as an ornamental plant but also in traditional medicine of Mediterranean areas, being extensively cultivated in Southern Europe [18]. It has an extremely variable chemotype, creating confusion between species.

Although some *Mentha* species were extensively studied, there is little information regarding the anti-inflammatory and antinociceptive properties of their essential oils, the major constituents. This original work is aimed at correlating the chemotype of three *Mentha* species from Romania with an in vivo study of the anti-inflammatory and antinociceptive effects, with the purpose of exploring potential benefits of essential oils in the treatment of various inflammatory conditions.

## 2. Results and Discussion

### 2.1. GC-MS Analysis of the Essential Oils

The GC-MS analysis of the essential oils isolated from the selected species of *Mentha* revealed the presence of over 30 compounds, mainly with terpenoid structures. Their retention indices and relative proportions in the studied samples are listed in Table 1.

Thus, in the essential oil from *M. piperita pallescens* (EOMPA), the major compounds were menthol (39.695% ± 3.26%), menthone (15.742% ± 2.73%), and isomenthone (7.735% ± 2.03%). Our data also showed the presence of estragole (0.929% ± 0.18%) in EOMPA, but the low content complies with the European Medicines Agency recommendations regarding the use of herbal medicinal products containing estragole [19]. In the essential oil from *M. spicata* (EOMSP) carvone (41.215% ± 4.18%) was the major compound, followed by menthol (12.774% ± 2.48%), menthone (7.225% ± 1.81%), and pulegone (3.763% ± 1.04%). In the essential oil from *M. suaveolens* (EOMSU) piperitenone oxide (73.773% ± 6.41%) was the major compound followed by germacrene-d (3.309% ± 1.19%) and limonene (2.969% ± 1.02%).

In this study, the GC-MS analysis of the essential oils from the tested samples showed a menthol chemotype for *M. piperita pallescens* cultivated in Romania, confirming the study of Schmidt et al., which found a rather similar menthol content (40.7%) in *M. piperita* originating from the Balkans [20].

Although our data confirmed a carvone chemotype for *M. spicata*, the carvone content was lower compared to *M. spicata* from India, where it reached 76.65% [21], or Turkey, where it reached 80.65% [22].

Our study showed also that a low content of carvone (1.555%) coupled with a high content of piperitenone oxide (73.773%), suggest a piperitenone oxide chemotype for *M. suaveolens*, encountered also in Southeastern Europe [23]. A different study (El-Kashouri et al.) found a higher percentage of carvone (24.72%–55.74%) in *M. suaveolens* harvested from North Africa [24]. The variations in chemical composition of essential oils from *Mentha* species can be attributed to several factors such as temperature, humidity, climate, harvest season, or photoperiod [25].

### 2.2. Anti-Inflammatory Activity of the Essential Oils

The anti-inflammatory activity of the tested essential oils was evaluated in vivo by the rat paw edema test induced by λ-carrageenan. The results of the experiment are presented in Table 2.

A statistically significant anti-inflammatory effect was observed in the groups treated with essential oils from *M. spicata* (EOMSP) 500 mg/kg, 2 h, 3 h, and 4 h after the induction of inflammation and *M. piperita pallescens* (EOMPA) 500 mg/kg, 4 h after the induction of inflammation. The most active sample, EOMSP (500 mg/kg), was superior to the reference drug diclofenac, 2 h and 4 h after the induction of inflammation. The essential oil from *M. suaveolens* (EOMSU) produced an inferior anti-inflammatory effect, and only at the highest dose.

The development of edema in the rat hindpaw following an injection of λ-carrageenan has been characterized as a biphasic event. Initially, the inflammatory reaction to carrageenan (0–1 h) is caused by the release of histamine, serotonin, bradykinin, complement and reactive oxygen species. In the second, accelerating the phase of swelling (2–4 h), an increased production of prostaglandins in the inflammatory area has been demonstrated [26].

Our experimental data suggest that several peripheral mechanisms could be responsible for the anti-inflammatory effect of essential oils from *Mentha*. Besides a reduction of prostaglandin concentration in the affected tissue, it is possible that the essential oils were able to also influence the first phase of carrageenan-induced edema formation, probably by inhibiting the release of other pro-inflammatory mediators.

### 2.3. Antinociceptive Activity of the Essential Oils

#### 2.3.1. Acetic Acid Induced Writhing Test in Mice

The antinociceptive activity of the tested essential oils was firstly evaluated by the acetic acid induced writhing test in mice. The results of the experiment are presented in Table 3.

The administration of the reference drug, diclofenac, significantly reduced the number of writhes induced by acetic acid, the percentage of inhibition being 60.49%. The essential oil from *M. spicata* (EOMSP) showed a significant and dose-dependent antinociceptive effect, reducing the number of writhes at all the three tested doses, although the results were slightly inferior to the reference drug, diclofenac. The essential oils from *M. piperita pallescens* (EOMPA) and *M. suaveolens* (EOMSU) showed inferior antinociceptive effects in this experimental model.

Acetic acid is known to trigger an irritative reaction in the peritoneum, which induces the writhing response due to the sensitization of nociceptors by prostaglandins, excessively formed in the peritoneal cavity. The nociceptive properties of acetic acid might also be due to the release of cytokines, such as TNF-α, interleukin-1β, and interleukin-8, by resident peritoneal macrophages and mast cells [27]. Thus, the abdominal constriction response induced by acetic acid is a sensitive procedure to establish peripherally acting antinociceptives [28].

The protective effect of essential oils against the chemical noxious stimulus may be an indication for a decreased production of prostaglandins, thereby causing a reduction in the number of writhes. Our results from this experimental model indicate that antinociceptive effect of EOMSP might be mediated by the peripheral inhibition of prostaglandins synthesis or actions.

#### 2.3.2. Hot Plate Test in Mice

To evaluate whether the antinociceptive effect of essential oils from *Mentha* might posses also a central mechanism, the hot plate test was used, the results being presented in Table 4.

The mice treated with EOMSP presented the most significant antinociceptive effects in the hot plate test, starting 60 min after the administration, with a peak after 120 min, at the dose of 500 mg/kg. For this group, the percent analgesic score (PAS) varied between 60.24% at 60 min and 65.40% at 120 min. The antinociceptive effects of EOMPA were slightly inferior but significant at 60, 90, and 120 min, at the dose of 500 mg/kg. EOMSU produced only modest results. The effects of morphine, the reference centrally acting antinociceptive drug, were evident 60 min after administration, peaked at 90 min and continued for 2 h. The results showed that the essential oils had a rapid effect, which peaked at 120 min, probably due to specific pharmacokinetic parameters.

In our tested essential oils samples, terpenoids were the most important molecules, being very diverse from a structural point of view. Thus, in the essential oil from *M. piperita pallescens* (EOMPA), menthol, a monocyclic alcohol was the predominant constituent. In the essential oil from *M. spicata* (EOMSP), carvone, a monoterpene with ketonic function, was the majoritary compound, while in *M. suaveolens* (EOMSU), piperitenone-oxide, also a monoterpene, but with an epoxy group, was the main active constituent. The functional groups of these terpenic essential oil constituents can influence not only their chemical properties but also the pharmacological interactions with their molecular targets [29].

According to Galeotti et al., the antinociceptive effect of the essential oils in the hot plate test can be partially attributed to the high content of menthol which can directly stimulate opioid receptors, (-)-menthol having superior analgesic effects [30]. Additionally, Gaudioso et al. found that menthol can also produce a use-dependent block of the Na^+^ channels from the dorsal root ganglion neurons with a subsequent pain modulation [31]. Although these studies indicate a central mechanism of action, Sun et al. suggested also that menthol, present in high concentrations in the essential oil from *M. piperita* grown in China, may inhibit PGE2 production with potent anti-inflammatory effects in vitro and in vivo [32].

Carvone, another important component present in the essential oils extracted from *Mentha* species, act mainly by peripheral mechanisms with a reduction of prostaglandin synthesis and inhibition of Nf-κB intracellular signaling, with subsequent anti-inflammatory and antinociceptive effects [33]. De Sousa et al. found that carvone and pulegone showed superior antinociceptive effects in the acetic acid-induced writhing test in mice, compared to piperitenone-oxide (rotundifolone) [34]. In another study, piperitenone-oxide was tested for its antinociceptive effects at doses of 10, 100, and 200 mg/kg, in the hot plate, writhing and tail-flick test, producing moderate effects only at the highest doses [35]. The majority of the experimental models used to study the anti-inflammatory and antinociceptive effects of terpenoids focused on acute inflammation, further research being needed to ascertain the validity of these findings in chronic inflammatory processes.

Our experimental study showed that the most significant anti-inflammatory and antinociceptive effects were produced by the essential oil from *M. spicata* (EOMSP), followed by the essential oil from *M. piperita* (EOMPA) and the essential oil from *M. suaveolens* (EOMSU) which showed only modest effects due to a different chemical composition.

According to our data, a high content of carvone and menthol could be responsible for the anti-inflammatory and antinociceptive effects, the amplitude of these effects depending on their concentration in the essential oil samples. However, due to the fact that essential oils are complex mixtures containing also other classes of molecules, it is possible that the pharmacological activity can be modulated by other minor components. Further research is needed to clarify the molecular mechanism of action of essential oils.

## 3. Materials and Methods

### 3.1. Plant Material and Essential Oil Isolation

The selected Mint species were *Mentha x piperita* L. var. *officinalis* Sole f. *pallescens* Camus (white peppermint), *Mentha spicata* L. subsp. *crispata* (spearmint), and *Mentha suaveolens* Ehrh. var. *variegata* (pineapple mint).

The plants were harvested in flowering phase from experimental fields of the Fares BioVital Laboratories Orastie (Hunedoara County, Romania, Latitude: 45°49.9998′ N Longitude: 23°12′ E) in July 2015. The experimental cultures of the three *Mentha* species were created in a randomized complete block design with four replications. The plants were planted 10 cm apart in 70 cm rows to rows, in a clay sandy soil. The plants received normal inter-cultural operations and irrigation. The average day temperature during summer season was 20 °C. After harvesting, voucher specimens (No. 1516, No. 1517 and No. 1518) were deposited in the Herbarium of the Department of Pharmaceutical Botany from the Faculty of Pharmacy, “Iuliu Hatieganu” University of Medicine and Pharmacy, Cluj-Napoca, Romania. For the isolation of essential oils, fresh leaves from each of the selected *Mentha* species were placed in a Clevenger apparatus and submitted to a hydrodistillation for 3 h. The yield of essential oil extraction was 0.98 mL/100 g herbal product for *Mentha piperita pallescens*, 1.03 mL/100 g herbal product for *Mentha spicata*, and 0.87 mL/100 g herbal product for *Mentha suaveolens*. Anhydrous sodium sulfate was added to the resulting organic phase in order to eliminate water traces, and the essential oils were then stored in sealed dark glass recipients at 4 °C. The GC-MS analysis and biological tests were performed on samples from the same batch for each of the tested *Mentha* species.

### 3.2. GC-MS Analysis of the Essential Oils

Qualitative and quantitative analysis of the essential oils isolated from the selected *Mentha* species was performed by gas chromatography coupled with mass spectrometry [36]. The gas chromatograph (Agilent Technologies model 7890A, Santa Clara, CA, USA) was equipped with a HP-5MS capillary column (phenyl-methyl-siloxane30 m × 250 µm × 0.25 µm, Agilent 19091S-433). Each diluted essential oil sample (1/100 in *n*-hexane, *v*/*v*) was injected in 1 µL volume, in split mode. The flow rate was 16 mL/min and the split ratio was 1:15. The oven temperature was linearly programmed from 60 °C to 240 °C (at rate of 3 °C/min) and then held for 10 min at the last temperature. The carrier gas was helium with a head pressure of 8.2317 psi. The mass spectrometer (Agilent Technologies model 5975C) operated in electron impact (EI) mode at 70 eV with an ion source temperature set at 250 °C. Mass spectra were acquired with the detector operating in scan mode, in 50–550 *m*/*z* range.

The retention index (RI) was calculated for all the volatile constituents using an *n*-alkane homologous series, using a linear temperature programmed equation [37]. The identification of essential oil components was performed by comparison of retention indices and mass spectra fragmentation patterns with the Willey and NIST database (6th ed.) as previously described [38]. ChemStation software (Agilent Technologies) was used for data analysis. The relative proportion of each individual component (%) was expressed as percent peak area relative to total peak area from the GC-MS analysis of the whole sample. The relative proportions of the components were presented as mean ± SD of three GC-MS analysis of each sample.

### 3.3. Animals

For the pharmacological experiments, 11 groups of male Charles River Wistar (Crl:WI) rats (*n* = 6) with a mean weight of 200 g and 22 groups of male Swiss albino mice (*n* = 6) with a mean weight of 30 g were obtained from the Practical Skills and Experimental Medicine Centre of the Iuliu Haţieganu University of Medicine and Pharmacy Cluj-Napoca (Romania). The animals were housed in polycarbonate type IV-S open-top cages (Tecniplast, Italy) and maintained under standard conditions (22 ± 2 °C, a relative humidity of 45% ± 10%, 12:12-h light:dark cycle). The animals had access to a standard pelleted feed (Cantacuzino Institute, Bucharest, Romania) and filtered water *ad libitum* throughout the experiment, except for the day when the test substances were administered. All experimental protocols were approved by the Ethics Committee of the Iuliu Hatieganu University of Medicine and Pharmacy, Cluj-Napoca, Romania, and were conducted in accordance with the EEC Directive 63/2010, which regulates the use of laboratory animals for scientific purposes.

### 3.4. Anti-Inflammatory Activity

The anti-inflammatory activity of the essential oils from the selected species of *Mentha* was determined by the rat paw edema test induced by λ-carrageenan, according to the method of Winter et al. modified by Griesbacher et al. after the introduction of a commercially available plethysmomether [39,40,41]. Thus, the essential oils from *M. piperita pallescens* (EOMPA), *M. spicata* (EOMSP), and *M. suaveolens* (EOMSU), after being solubilized in the vehicle (1% Tween 80 aqueous solution), were orally administered to 9 groups of Crl:WI rats (*n* = 6) in three different doses—125 mg/kg bw, 250 mg/kg bw, and 500 mg/kg bw—one hour before the induction of inflammation. Rats in the control group were orally treated with the vehicle (10 mL/kg), while rats in the reference group received, also orally, 20 mg/kg bw diclofenac (Gerot Lannach GmbH, Lannach, Austria), a non-steroidal anti-inflammatory drug. Inflammatory edema was induced by a single injection of 0.1 mL of 1% λ-carrageenan (Sigma Aldrich, St. Louis, MO, USA) into the subplantar region of the left hind paw of each rat. The paw volume of each animal was determined before carrageenan injection and at 1, 2, 3, and 4 h after the induction of inflammation with a digital plethysmometer (model 7140, Ugo Basile, Varese, Italy). Edema volume and the percentage of edema inhibition were calculated as follows:

Edema volume (mL) = Vt − Vo

Inhibition of edema (%) = [1− (Et/Ec) × 100]

where Vo is the mean paw volume before carrageenan injection, Vt is the mean paw volume at “t” hours, Et is mean edema volume in treated animals, and Ec is mean edema volume in the control group.

### 3.5. Antinociceptive Activity

#### 3.5.1. Acetic Acid Induced Writhing Test

The antinociceptive effect of the essential oils from the selected species of *Mentha* was evaluated by the writhing test in mice, using 1% (*v*/*v*) acetic acid solution administered intraperitonealy to induce abdominal constrictions [42]. Initially, the essential oils from *M. piperita pallescens* (EOMPA), *M. spicata* (EOMSP), and *M. suaveolens* (EOMSU), after being solubilized in the vehicle (1% Tween 80 aqueous solution), were orally administered to 9 groups of male Swiss mice (*n* = 6) in three different doses: 125 mg/kg bw, 250 mg/kg bw, and 500 mg/kg bw, orally, by gastric intubation. The mice in the control group (*n* = 6) were treated orally with the vehicle (10 mL/kg). The animals from the reference group (*n* = 6) were treated orally with an anti-inflammatory drug, diclofenac 20 mg/kg bw. After 30 min, all mice were injected intraperitoneally with 0.1 mL of 1% acetic acid solution, in order to induce abdominal constrictions (writhes). The animals were placed in an observation box, the writhes being counted over a period of 20 min. For scoring purposes, a writhe was indicated by stretching of the abdomen with simultaneous stretching of at least one hind limb. The analgesic activity was evaluated by calculating the percentage of inhibition of the writhes with the formula:

% inhibition = (mean no. of writhes for control group − mean no. of writhes for treated group) × 100/mean no. of writhes for control group.

#### 3.5.2. Hot Plate Test in Mice

The antinociceptive effect of the essential oils from the selected species of *Mentha* was also evaluated by the hot plate test in mice [43], using a digital Hot/Cold Plate (model 35100, Ugo Basile Varese, Italy). Each mouse was initially placed on the plate heated at 55 °C in order to observe its pain responses (hind paw licking or jumping). The time (in seconds) needed for the development of this pain response was recorded by the device for each individual animal. The mice exhibiting response times shorter than 5 s or longer than 30 s were excluded from the study. Afterwards, the essential oils from *M. piperita pallescens* (EOMPA), *M. spicata* (EOMSP), and *M. suaveolens* (EOMSU), after being solubilized in the vehicle (1% Tween 80 aqueous solution), were orally administered to 9 groups of male Swiss mice (*n* = 6) in three different doses: 125 mg/kg bw, 250 mg/kg bw, and 500 mg/kg bw, orally, by gastric intubation. The mice in the control group (*n* = 6) were treated orally with the vehicle (10 mL/kg). The animals from the reference group (*n* = 6) were treated orally with a centrally acting antinociceptive drug, morphine, at 10 mg/kg bw. The evaluation of the response times was repeated for each individual animal at 30 min, 60 min, 90 min, and 120 min from the substance administration.

Percent analgesic score (PAS) was calculated for each group at all time intervals as:

PAS = (Tf − Ti)/Tf × 100

where Tf = response time (in seconds) after drug administration, and Ti = response time (in seconds) before drug administration.

### 3.6. Statistical Analysis

Data were expressed as mean values ± SD and were statistically analyzed by one-way ANOVA method. The differences between the treated groups and the control group were evaluated by Dunnett’s *t*-test, *p*-values ≤ 0.05 being considered statistically significant.

## 4. Conclusions

This research showed a menthol chemotype for *M. piperita pallescens*, a carvone chemotype for *M. spicata* and a piperitenone oxide chemotype for *M. suaveolens*, cultivated in Romania. Our experimental results showed that the essential oil from *M. spicata* L. (EOMSP) with the particular chemical composition presented in the study had significant and dose-dependent anti-inflammatory and antinociceptive properties. Since the essential oil composition can be influenced by a variety of factors, further research is necessary to demonstrate if these promising results can be extrapolated to a wider variety of *M. spicata* samples, under different environmental conditions.

## Figures and Tables

**Table 1:** Chemical composition of the essential oils from the selected *Mentha* species.

No.	Compound	RI_lit_^a^	RI_cal_^b^	% in EOMPA ^c^	% in EOMSP ^d^	% in EOMSU ^e^
1	alpha-pinene	939	950	0.133 ± 0.03	0.220 ± 0.05	0.791 ± 0.12
2	sabinene	971	980	0.071 ± 0.00	0.136 ± 0.03	0.342 ± 0.08
3	beta-pinene	976	983	0.232 ± 0.04	0.471 ± 0.19	1.497 ± 0.42
4	myrcene	990	994	0.119 ± 0.02	0.095 ± 0.00	0.462 ± 0.11
5	2-octanol	997	997	0.617 ± 0.18	0.201 ± 0.06	0.305 ± 0.07
6	para-cymene	1022	1025	0.292 ± 0.11	0.140 ± 0.02	0.230 ± 0.05
7	limonene	1027	1028	0.346 ± 0.09	1.569 ± 0.48	2.969 ± 1.02
8	1,8-cineole	1029	1031	1.587 ± 0.64	2.567 ± 0.69	0.118 ± 0.04
9	gamma-terpinene	1057	1058	0.407 ± 0.05	0.106 ± 0.00	-
10	menthone	1151	1155	15.742 ± 2.73	7.225 ± 1.81	-
11	isomenthone	1162	1165	7.735 ± 2.03	3.325 ± 0.82	-
12	menthol	1170	1177	39.695 ± 3.26	12.774 ± 2.48	0.128 ± 0.03
13	terpineol 4	1175	1179	2.182 ± 0.55	1.221 ± 0.33	0.679 ± 0.19
14	isomenthol	1181	1184	0.493 ± 0.14	0.192 ± 0.04	-
15	alpha-terpineol	1188	1191	0.449 ± 0.17	0.622 ± 0.21	0.246 ± 0.04
16	dihydrocarveol	1192	1195	0.120 ± 0.02	1.120 ± 0.24	-
17	estragole	1195	1199	0.929 ± 0.18	-	-
18	*trans*-carveol	1216	1219	0.085 ± 0.00	1.113 ± 0.47	-
19	*cis*-carveol	1228	1232	-	1.221 ± 0.31	-
20	pulegone	1235	1239	2.140 ± 0.80	3.763 ± 1.04	-
21	carvone	1240	1244	2.377 ± 0.73	41.215 ± 4.18	1.555 ± 0.44
22	piperitone	1250	1254	2.096 ± 0.75	0.647 ± 0.13	0.340 ± 0.08
23	neomenthyl acetate	1274	1276	0.135 ± 0.02	0.101 ± 0.00	-
24	trans-anethole	1282	1286	5.374 ± 0.94	0.113 ± 0.02	-
25	menthyl-acetate	1294	1295	3.022 ± 1.21	1.912 ± 0.89	-
26	menthylcamphor	-	1300	0.478 ± 0.17	0.497 ± 0.12	0.498 ± 0.15
27	eugenol	1354	1357	0.214 ± 0.05	0.184 ± 0.03	-
28	piperitenone oxide	1366	1366	-	-	73.773 ± 6.41
29	beta-bourbonene	1383	1384	0.222 ± 0.04	0.951 ± 0.27	0.293 ± 0.05
30	cis-jasmone	1395	1399	-	-	2.124 ± 0.65
31	caryophyllene	1418	1418	0.112 ± 0.03	2.289 ± 0.99	0.604 ± 0.23
32	germacrene-d	1479	1480	-	-	3.309 ± 1.19
33	viridoflorol	1592	1590	-	-	1.455 ± 0.68

^a^ Retention indices from literature. ^b^ Calculated retention indices. ^c^ EOMPA: essential oil from *M. piperita pallescens*. ^d^ EOMSP: essential oil from *M. spicata*. ^e^ EOMSU: essential oil from *M. suaveolens*. Relative proportions are expressed as mean ± SD of three GC-MS analysis of each sample.

**Table 2:** Effect of the essential oils from the selected *Mentha* species on carrageenan-induced rat paw edema.

Group	Dose	Edema 1 h (mL)	Edema 2 h (mL)	Edema 3 h (mL)	Edema 4 h (mL)
		(% inhib.)	(% inhib.)	(% inhib.)	(% inhib.)
Control (vehicle)	-	0.56 ± 0.11	1.30 ± 0.13	2.00 ± 0.20	2.34 ± 0.27
EOMPA	500 mg/kg	0.36 ± 0.20	0.96 ± 0.40	1.40 ± 0.64	1.12 ± 0.84 *
		(35.71%)	(25.15%)	(30.00%)	(52.13)
EOMPA	250 mg/kg	0.42 ± 0.04	1.11 ± 0.53	1.69 ± 0.73	1.80 ± 0.46
		(25.00%)	(14.61%)	(15.50%)	(23.07%)
EOMPA	125 mg/kg	0.50 ± 0.15	1.21 ± 0.66	1.81 ± 0.98	2.03 ± 1.18
		(10.71%)	(6.92%)	(9.50%)	(13.24%)
EOMSP	500 mg/kg	0.36 ± 0.17	0.52 ± 0.28 *	0.74 ± 0.26 *	0.88 ± 0.26 *
		(35.71%)	(60.00%)	(63.00%)	(62.39%)
EOMSP	250 mg/kg	0.44 ± 0.27	0.78 ± 0.19 *	1.23 ± 0.46	1.54 ± 0.86
		(21.42%)	(40.00%)	(38.50%)	(34.18%)
EOMSP	125 mg/kg	0.50 ± 0.37	1.03 ± 0.69	1.68 ± 1.13	2.01 ± 0.64
		(10.71%)	(20.76%)	(16.00%)	(14.10%)
EOMSU	500 mg/kg	0.54 ± 0.35	0.98 ± 0.64	1.23 ± 0.89	1.41 ± 0.78
		(3.57%)	(24.61%)	(38.50%)	(39.74%)
EOMSU	250 mg/kg	0.63 ± 0.22	1.20 ± 0.80	1.56 ± 0.73	1.84 ± 0.98
		(-)	(7.69%)	(22.00%)	(21.36%)
EOMSU	125 mg/kg	0.70 ± 0.31	1.42 ± 0.69	1.79 ± 0.46	2.13 ± 1.36
		(-)	(-)	(10.5%)	(8.9%)
Diclofenac	20 mg/kg	0.36 ± 0.06 *	0.55 ± 0.11 *	0.68 ± 0.08 *	1.06 ± 0.17 *
		(35.71%)	(57.69%)	(66.00%)	(54.70%)

* Statistically significant, *p* ≤ 0.05. Values are expressed as Mean ± SD.

**Table 3:** Effect of the essential oils from the selected *Mentha* species in the acetic acid induced writhing test in mice.

Group	Dose (mg/kg)	No. of Writhes (X ± SD)	Percentage of Inhibition (%)
Control (vehicle)	-	32.4 ± 10.19	-
EOMPA	500 mg/kg	20.8 ± 4.25	35.80
EOMPA	250 mg/kg	23.6 ± 7.22	27.16
EOMPA	125 mg/kg	25.6 ± 5.15	20.98
EOMSP	500 mg/kg	17 ± 7.87 *	47.53
EOMSP	250 mg/kg	18.8 ± 9.23 *	41.97
EOMSP	125 mg/kg	19.8 ± 6.60 *	38.88
EOMSU	500 mg/kg	24.2 ± 2.56	25.30
EOMSU	250 mg/kg	28 ± 2.89	13.58
EOMSU	125 mg/kg	30.6 ± 2.05	5.55
Diclofenac	20 mg/kg	12.8 ± 4.12 *	60.49

* Statistically significant, *p* ≤ 0.05. Values are expressed as Mean ± SD.

**Table 4:** Effect of the essential oils from the selected *Mentha* species in the hot plate test in mice.

Group	Response (s) at 0 min (PAS)	Response (s) at 30 min (PAS)	Response (s) at 60 min (PAS)	Response (s) at 90 min (PAS)	Response (s) at 120 min (PAS)
Control (Vehicle)	8.78 ± 2.18	9.12 ± 1.94	8.19 ± 1.53	7.85 ± 1.80	7.13 ± 1.96
	(-)	(-)	(-)	(-)	(-)
EOMPA 500 mg/kg	9.05 ± 1.76	15.89 ± 2.27	20.98 ± 6.17 *	23.44 ± 4.72 *	25.12 ± 8.47 *
	(-)	(43.04%)	(56.86%)	(61.39%)	(63.97%)
EOMPA 250 mg/kg	10.34 ± 2.20	13.44 ± 3.67	17.39 ± 4.99 *	19.89 ± 4.63	21.27 ± 5.46 *
	(-)	(23.06%)	(40.54%)	(48.01%)	(51.38%)
EOMPA 125 mg/kg	9.42 ± 1.96	11.29 ± 2.16	14.52 ± 4.21	17.11 ± 3.92	18.41 ± 4.52
	(-)	(16.56%)	(35.12%)	(44.94%)	(48.83%)
EOMSP 500 mg/kg	9.84 ± 1.98	18.23 ± 3.76	24.75 ± 6.71 *	27.42 ± 6.06 *	28.44 ± 4.77 *
	(-)	(46.02%)	(60.24%)	(64.11%)	(65.40%)
EOMSP 250 mg/kg	10.31 ± 0.84	14.97 ± 3.70	19.42 ± 4.63	22.93 ± 6.35 *	24.03 ± 6.15 *
	(-)	(31.12%)	(46.91%)	(55.03%)	(57.09%)
EOMSP 125 mg/kg	9.24 ± 1.87	12.19 ± 3.90	15.55 ± 3.09 *	18.32 ± 4.57 *	20.84 ± 5.82 *
	(-)	(24.20%)	(40.57%)	(49.56%)	(55.66%)
EOMSU 500 mg/kg	10.11 ± 3.09	13.42 ± 3.23	18.11 ± 2.04	20.75 ± 4.54 *	21.04 ± 6.64 *
	(-)	(24.66%)	(44.17%)	(51.27%)	(51.94%)
EOMSU 250 mg/kg	9.19 ± 1.96	11.41 ± 2.74	15.14 ± 4.41	17.02 ± 5.15	16.14 ± 3.90
	(-)	(19.45%)	(39.29%)	(46.00%)	(43.06%)
EOMSU 125 mg/kg	10.04 ± 2.11	10.11 ± 1.76	13.45 ± 2.98	14.25 ± 4.50	13.89 ± 3.70
	(-)	(6.9%)	(25.35%)	(29.54%)	(27.71%)
Morphine 10 mg/kg	10.23 ± 2.92	11.50 ± 4.21	35.63 ± 7.58 *	38.72 ± 6.64 *	37.45 ± 6.77 *
	(-)	(11.04%)	(71.28%)	(73.57%)	(72.68)

* Statistically significant, *p* ≤ 0.05. Values are expressed as Mean ± SD.
